# The analgesic efficacy of oxycodone hydrochloride versus fentanyl during outpatient artificial abortion operation: A randomized trial

**DOI:** 10.1097/MD.0000000000007376

**Published:** 2017-06-30

**Authors:** Kangjie Xie, Wen Zhang, Wumei Fang, Yanhong Lian, Sufeng Lin, Jun Fang

**Affiliations:** a Department of Anesthesiology, Zhejiang Cancer Hospital; b Department of Anesthesiology, Maternal and Child Health Hospital of Yuhang District, Hangzhou, People's Republic of China.

**Keywords:** artificial abortion, oxycodone, pains of uterus systol

## Abstract

**Background:**

Problems like body movement, respiratory depression, and complained of pain are still common phenomenon in outpatient artificial abortion general anesthesia. Oxycodone hydrochloride is a semisynthetic opioid and has a good therapeutic effect on visceral pain. We hypothesize that oxycodone hydrochloride would be superior to fentanyl in outpatient artificial abortion surgery.

**Methods:**

In this clinical trial 149 American Society of Anesthesiologists (ASA) I or II female outpatients scheduled for elective artificial abortion surgeries under general anesthesia were randomly divided into 3 groups: oxycodone hydrochloride 0.06 mg/kg group (group A), oxycodone hydrochloride 0.08 mg/kg group (group B), and control group fentanyl 2 ug/kg (group C). The primary outcome was level body movement and respiratory depression during the surgery, the second outcome included the visual analogue scale (VAS) score 30 minutes after waking.

**Results:**

A total of 120 participants completed the study, n = 40 in each group. There was no significant difference in patients’ age, body mass index (BMI), preoperative heart rate, mean arterial blood pressure, consumption dose of propofol, intraoperative body movement type and times, and duration of surgery among the 3 groups (*P* > .05). Comparing the incidence of intraoperative respiratory depression and SPO2 < 90% among the 3 groups, group C's was significantly higher than those of groups A and B, and the difference was statistically significant (*P* < .05). Group A had no difference compared with group B. In VAS score 30minutes after waking, group C was the highest, followed by group A, with group B as the lowest. The difference among the 3 groups was statistically significant (*P* < .05), but a difference delta less than 1 on the VAS scale is not clinically significant.

**Conclusion:**

The analgesic effect of oxycodone hydrochloride at 0.06 mg/kg applied to painless artificial abortion surgery is not superior than that of fentanyl, but the incidence of intraoperative respiratory depression and hypoxemia is significantly lower than fentanyl.

## 1 Introduction

Oxycodone hydrochloride is a semisynthetic opioid and a μ and κ receptor agonist targeting the central nervous system and smooth muscle in its pharmacological effects. It is used for the treatment of acute and chronic pain in clinical applications.^[1,2]^ Published studies report that compared with other IV opioids for postsurgical pain, oxycodone is a safe and effective analgesic, IV oxycodone may be associated with greater pain control, fewer or less severe adverse events, and faster onset of action.^[3]^ Nonintubated general anesthesia is a widely used method in China for outpatient artificial abortion anesthesia, but many patients have body movement and respiratory depression during the surgery, or complained of pain after waking. Raeder's study shows that of the patients who received general anesthesia, 25% had apnea and 67% experienced postoperative pain.^[4]^ Propofol and fentanyl are commonly used drugs for nonintubated general anesthesia in our clinical work, but we still wrestle with problems such as body movement, respiratory depression, and complained of pain. In this study, we hypothesize that oxycodone hydrochloride would be superior to fentanyl in outpatient artificial abortion surgery. We evaluate the efficacy and safety of oxycodone hydrochloride for analgesia in outpatient artificial abortion and explore the optimal dose application.

## 2 Subjects and methods

### 2.1 Subjects

This prospective clinical study was conducted after receiving approval from the Human Research Ethics Board of Zhejiang Cancer Hospital, Hangzhou, and Maternal and Child Health Hospital of Yuhang District, Hangzhou. A signed written informed consent document was obtained from each patient prior to participation.

The inclusion criteria were American Society of Anesthesiologists (ASA) physical status I and II female patients aged 20 to 42 scheduled for outpatient artificial abortion operation and requiring general anesthesia. Exclusion criteria included hepatitis and renal failure, existence of severe cardiovascular or cerebrovascular disease, a history of chronic pain, or mental illness. Those allergic to oxycodone hydrochloride, fentanyl, or propofol and those with habitual sedative, analgesic, or other drug use were excluded from this study.

Subjects were randomly divided into 3 groups: a group using oxycodone 0.06 milligram per kilogram (mg/kg; group A), and a group using oxycodone 0.08 mg/kg (group B), a control group using fentanyl 2 micrograms per kilogram (ug/kg; group C). Both subjects and data collectors were blinded to group allocation except the attending anesthetist.

## 3 Methods

On day 1 of this study, demographic and medical data, including the subject's age, body mass index (BMI), and history of diseases, were collected. The patients were not given any sedative, analgesic, antemetic, or anti-itching drugs 24 hours before the operation. Patients were fasting from solids and liquids 8 hours before the operation.

On day 2, an 18-gauge cannula was inserted into a forearm vein, oxygen inhalation of 3 L per minute through a nasal tube was applied, and standard monitoring (electrocardiogram, pulse oximetry, and noninvasive blood pressure) was established prior to the induction of anesthesia in the outpatient operating room. Then, propofol 2 mg/kg along with analgesic was administered intravenously. Groups A and B were administered oxycodone 0.06 or 0.08 mg/kg separately, as described above, whereas group C was delivered 2 ug/kg fentanyl. If the patient did not fall asleep or had a body quiver during the operation, additional propofol of 0.5 to 1 mg/kg was administered. The body quiver could be divided into class A, where only limbs quivered, and class B, where the torso quivered. No other analgesia or antiemetic drugs were applied during the operation and recovery period. Once oxygen saturation fell to 90%, assisted ventilation by a facial mask with oxygen would be applied.

Arterial oxygen saturation (via pulse oximetry), heart rate (via electrocardiography), and blood pressure (via noninvasive automated sphygmomanometer) were measured before anesthesia induction and throughout the operation and recovery period. The dose of opioids, propofol, respiratory depression (defined as respiratory rate less than 8 breaths per minute), operating time, and awakening time of anesthesia were collected. The level of pain, sedation, nausea and vomiting, functional activities, and chill were also evaluated and recorded after recovery (0, 10, 20, 30 minutes).

The level of pain was measured at movement by the visual analogue scale (VAS). Patients drew a point on a line, with 0 cm indicating no pain and 10 cm unbearable pain.

The level of sedation was evaluated by the Ramsay score. A patient who was anxious and agitated, restless, or both had a score of 1; a patient cooperative, oriented, and tranquil a score of 2; a patient responsive to commands only a score of 3; a patient asleep with a brisk response to light glabellar tap or loud auditory stimulus a score of 4; a patient asleep with sluggish response to light glabellar tap or loud auditory stimulus a score of 5, and a patient asleep with no response to light glabellar tap or loud auditory stimulus a score of 6.

For the level of nausea and vomiting, a score from 0 to 10 was given. A score of 0 was given if the patient had no nausea or vomiting, and a score of 10 was given if the patient had severe nausea and vomiting.

Functional activity score (FAS) scores from 0 to 2 to evaluate functional activity. A score of 0 was given if the patient had no activity limitation, and a score of 2 was given if the patient had severe activity limitation. Severe activity limitation means the difficulty or need of assistance for basic activities everyone is expected to perform independently: washing, getting (un)dressed, feeding, getting in and out of bed, using the toilet.^[5]^

Muscle chill was assessed by a score from 0 to 3. Zero represented no muscle chilling, whereas 1 meant face and neck muscle fibrillation. If more than 2 sets of muscles were chilled, the score was 2, and whole-muscle tremor with bed shaking scored 3.

### 3.1 Statistical analysis

We use PASS 11.0 software for sample size analysis. A sample size of 37 in each group was determined to be required for a power of 0.90 and an a-value of 0.05. The primary outcome was level body movement and respiratory depression during the surgery, the second outcome included the VAS score 30 minutes after waking. Categorical data were assessed with the *χ*^2^ test. Normally distributed continuous variables were analyzed with 1-way analysis of variance. Non-normally distributed data were further analyzed using the Mann–Whitney or Kruskal–Wallis H test. Lastly, *P* values <.05 were considered statistically significant.

## 4 Results

The characteristics of the enrolled subjects are shown in Table 1. A total of 149 subjects were recruited between January 1, 2016, and August 31, 2016, of whom 120 subjects finally completed the study with their data being analyzed for the final results (n = 40 per group). There were no significant differences in age, BMI, comorbidities, preoperative heart rate, or mean arterial pressure (MAP) among subjects in groups A, B, and C.

**Table 1:**
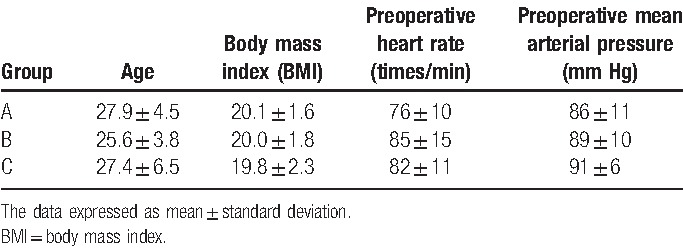
General comparison among group A, group B, and group C.

There were no statistically significant differences in the propofol initial dosage or added dosage, occurrence of body movement, incidence of MAP less than 70 mm Hg, or operation duration in the 3 groups. Two subjects emerged with bradycardia (defined as heart rate lower than 55 beats per minute) during operation in group C, whereas 4 subjects had bradycardia in group B, but there was no significant difference. Compared with groups A and B, group C had a higher percentage of respiratory depression and oxygen saturation lower than 90% (*P* < .05), whereas there was no difference between groups A and B. Also, there were no differences in awakening time of anesthesia or delayed awakening in the 3 groups (see Table 2).

**Table 2:**
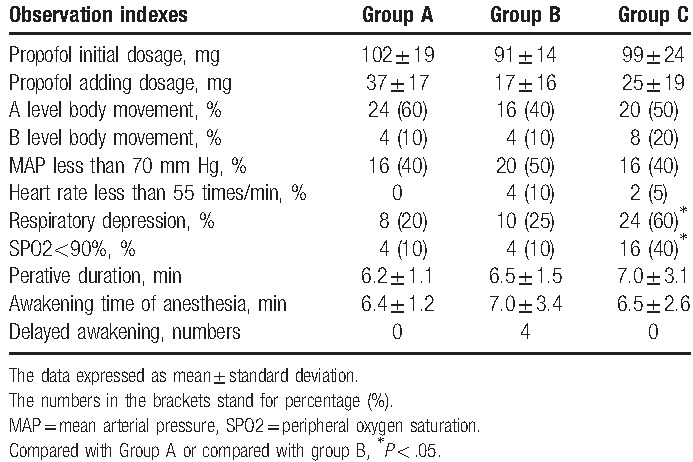
Comparison of group A, group B and group C during the operation.

As shown in Table 3, there were no statistically significant differences in the VAS pain score, nausea and vomiting, heart rate, MAP, or muscle chilling score among the 3 groups at awakening time. Group B had a higher Ramsay score than groups A and C (*P* < .05), whereas groups A and C showed no significant difference. Group B had the highest FAS, whereas group C had the lowest score (*P* < .05). Ten minutes after awakening time, the 3 groups showed no statistically significant differences in the VAS pain score, Ramsay score, nausea and vomiting, heart rate, MAP, muscle chilling score, or FAS. Twenty minutes after awakening, group C had a higher VAS pain score than group A (*P* < .05) even though no difference was found between the other groups. However, there were no differences in the Ramsay score, nausea and vomiting, heart rate, MAP, muscle chilling score, or FAS in the 3 groups. Additionally, group C had the highest VAS pain score, whereas group A had the lowest score 30 minutes after awakening (*P* < .05). Group C also had a higher MAP than group A, whereas there was no difference between groups A and B. But a difference delta less than 1 on the VAS scale is not clinically significant, we cannot conclude that the analgesic efficacy of groups A and B is better than group C.

**Table 3:**
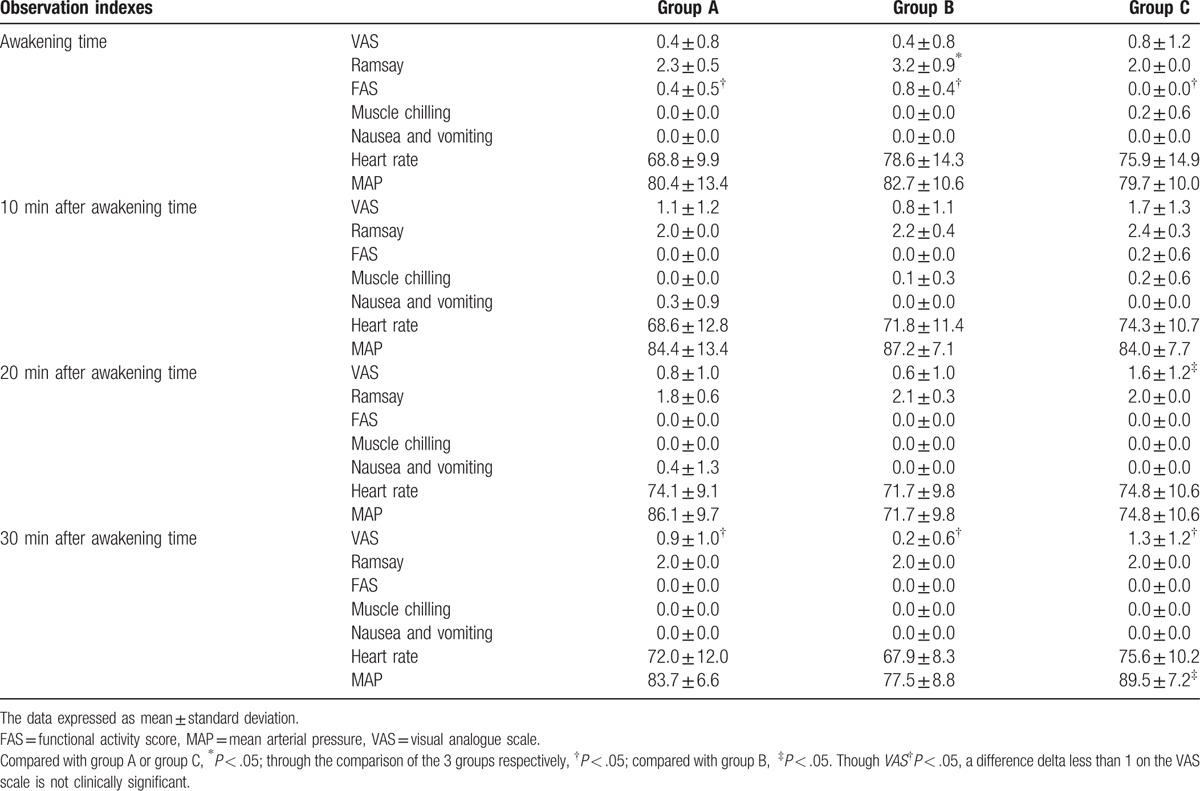
Comparison of 3 groups after operation.

## 5 Discussion

Heavy pain in muscle, viscera, and so on occurs frequently, which causes a significant challenge in clinical medicine. Opioid drugs are commonly used to treat heavy pain by targeting μ receptors. However, a number of adverse effects, such as inadequate analgesia, respiratory depression, and nausea, limit the application of opioids, particularly in visceral pain. Given the specific features of visceral pain, the κ opioid receptor that targets the intestinal peripheral nerve was recommended.^[1]^ Oxycodone hydrochloride is a semisynthetic opioid that is a μ and κ receptor agonist. Previous studies have shown that oxycodone hydrochloride has a good therapeutic effect on visceral pain.^[2]^ The artificial abortion operation mainly stimulates intraoperative pain and body movement and pains of uterus systole in the postoperative period. Propofol and fentanyl are widely used drugs for nonintubated general anesthesia in our clinical work, but we are still wrestle with problems like body movement, respiratory depression, and complained of pain. Clinical study proved that oxycodone showed comparable effects for pain relief compared with fentanyl in spite of less cumulative PCA dose.^[6]^ We hypothesize that oxycodone hydrochloride would be superior to fentanyl in outpatient artificial abortion surgery.

In this study, we investigated whether the efficacy and safety of oxycodone hydrochloride was superior to fentanyl in outpatient artificial abortion surgery, and examined the optimal dose of oxycodone hydrochloride. In the pretest, oxycodone hydrochloride and fentanyl were administered with equivalent doses, but oxycodone hydrochloride is a long-acting drug, which caused wake-up delay in patients and time-consuming turnover of outpatients after surgery. Therefore, we reduced the amount of oxycodone hydrochloride in the outpatient artificial abortion operations.

In our study, we found no statistically significant difference in the amount of propofol, body movement incidence, operative duration, or incidence of intraoperative hypotension among the 3 groups.

However, there were 4 cases of delayed awakening in group B. It shows 0.08 mg/kg dose of oxycodone hydrochloride is over dose for some patients. Previous studies have shown that the pharmacokinetics of oxycodone hydrochloride have individual differences, which are influenced by gender, age, and function of the liver and kidneys.^[7]^ Delayed awakening may also be related to differences in individual drug metabolism.

Concerning the incidence of respiratory depression and the incidence of SPO2 < 90% among the 3 groups, group C's incidences were significantly higher than the 2 different doses of oxycodone hydrochloride (groups A and B). There was no difference between group A and group B, indicating that for painless anesthesia under the same dose of propofol, fentanyl is more prone to respiratory depression and hypoxemia than oxycodone hydrochloride. Haji's study in decerebrate cats shows that the activation of κ receptors by itself depresses the central respiratory activity, while it opposes the μ receptor-mediated respiratory depression.^[8]^ This may explain why oxycodone hydrochloride have less respiratory depression than fentanyl. But we want to emphasize oxycodone hydrochloride do have respiratory depression even though it is less than fentanyl. A study shows that ethanol together with oxycodone causes greater ventilatory depression than either alone, the magnitude of which is clinically relevant.^[9]^ However, there was no difference in body movement between fentanyl (group C) and oxycodone hydrochloride (groups A and B), indicating that the analgesic effect in the 3 groups was similar.

There were 4 cases of delayed awakening in group B, but the operative effect in group B was not better than those of group A and group C, indicating that high doses of oxycodone hydrochloride do not produce better clinical effect, but may cause wake-up delay and affect the turnover of outpatient surgery. The Ramsay sedation score of group B was significantly higher than the other 2 groups. Group A's score was slightly higher than group C's, but there was no significant difference between the 2 groups, indicating that the sedative effect of oxycodone hydrochloride is better than fentanyl, with the feature of increased drowsiness. Kim et al^[10]^ demonstrate oxycodone could provide superior analgesia to fentanyl but higher incidence of side effects, including PONV, dizziness, and drowsiness. Hao et al^[11]^ have also shown that side effects of oxycodone hydrochloride include dizziness, nausea, vomiting, drowsiness, and fatigue. For the functional evaluation scores of the 3 groups, group C was the lowest, group A followed, and group B was the highest. However, there was no statistically significant difference among the 3 groups at 20 and 30 minutes after awakening, the fentanyl VAS score was significantly higher than those of the other 2 groups, indicating that oxycodone hydrochloride is better than fentanyl for the treatment of postoperative uterine pain and has a working time longer than that of fentanyl. But a difference delta less than 1 on the VAS scale is not clinically significant, so we cannot draw the conclusion that oxycodone hydrochloride is superior than fentanyl. Previous studies suggest that the peripheral nerve κ opioid receptors in the visceral pain system may play an important role in the analgesic effect.^[12]^ Previous studies have shown that oxycodone has an additional effect on κ opioid receptors to some extent; the analgesic effect may be mediated by κ opioid receptors.^[13]^ Thirty minutes after awakening, the VAS score of group B was significantly lower than that of group C, which indicated that the high-dose oxycodone hydrochloride had a longer analgesic effect. Thirty minutes after awakening, the MAP of the fentanyl group was significantly higher than that of group B, probably due to its short analgesic effect and the high blood pressure caused by a low analgesic effect.

Study limitations include a moderate number of enrolled patients, a short period of observation (30 minutes), and have no serum oxycodone/fentanyl assays. However, despite the aforementioned limitations, we were able to demonstrate an effective analgesic effect, good profile of adverse effects, of oxycodone intravenous injection at a dose of 0.06 and 0.08 mg/kg together with propofol 2 mg/kg for outpatient artificial abortion surgery. We also show oxycodone's advantage and disadvantage compared with fentanyle in the administration of outpatient artificial abortion anesthesia.

In summary, oxycodone hydrochloride can be a safe and effective application in the outpatient artificial abortion operation. The analgesic effect of oxycodone hydrochloride at 0.06 mg/kg applied to painless artificial abortion surgery is not superior than that of fentanyl, but the incidence of intraoperative respiratory depression and hypoxemia is significantly lower than fentanyl. The effect on oxycodone hydrochloride 0.08 mg/kg was not better than that on 0.06 mg/kg, but adverse effects were increased.
